# Heat Stress, Starvation, and Heat Stress Plus Starvation Cause Unique Transcriptomic Responses in the Economically Important Red Abalone *Haliotis rufescens*

**DOI:** 10.17912/micropub.biology.001473

**Published:** 2025-01-24

**Authors:** Hanna L. Franklin, Lani U. Gleason

**Affiliations:** 1 Department of Biological Sciences, California State University, Sacramento

## Abstract

Although most marine invertebrates are experiencing multiple environmental stressors simultaneously, the transcriptome-wide gene expression responses to multiple stressors remain understudied. We used RNA-sequencing to assess the transcriptomic responses to heat stress, starvation, and heat stress plus starvation in the red abalone *Haliotis rufescens.* Results indicate that the response to each stressor is distinct and is characterized by unique gene functions. The heat stress plus starvation treatment produced the largest transcriptomic response, including a significant upregulation of genes involved in translation. Overall, this study highlights the importance of multi-stressor experiments that reflect the complex modalities of climate change.

**Figure 1. Comparison of H. rufescens transcriptomic responses to experimental conditions:**
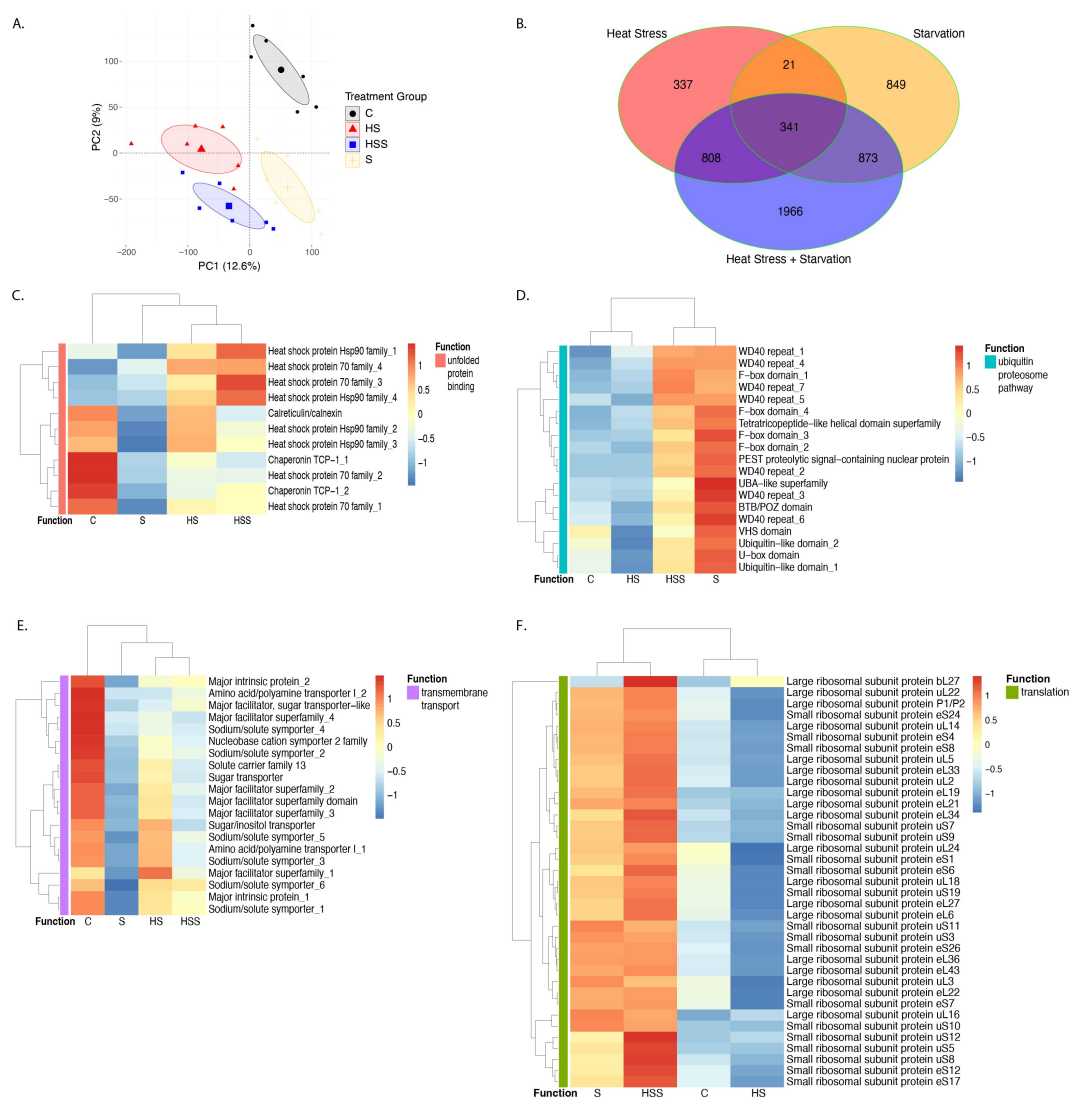
(A) Principal component analysis (PCA) scores on Principal Component 1 (PC1) and Principal Component 2 (PC2) of expression values for 45,965 total genes across four treatments. Ellipses represent 95% confidence intervals for each treatment. Numbers in parentheses on each axis indicate the proportion of variance explained by that Principal Component. Colors represent different treatment groups; each symbol represents one abalone. C, control, black, *n* = 6; HS, heat stress, red, *n* = 6; HSS, heat stress + starvation, blue, *n* = 6; S, starvation, yellow, *n* = 6. (B) Venn diagram depicting the number of differentially expressed genes in each treatment group when compared to the control group. Colors represent different treatment groups: Heat Stress, red; Heat Stress + Starvation, blue; Starvation, yellow. (C-F) Heat maps of the C) 11 unfolded protein binding genes that are upregulated in the heat stress and heat stress + starvation treatments and/or downregulated in the starvation treatment (indicated with the pink “Function” color); D) 19 genes in the ubiquitin proteosome pathway that are upregulated in the starvation and heat stress + starvation treatments (indicated with the teal “Function” color); E) 20 transmembrane nutrient transport genes that are significantly downregulated only in the starvation treatment (indicated with the purple “Function” color); and F) 37 translation genes that are significantly upregulated only in the heat stress + starvation treatment (indicated with the green “Function” color). Each heat map column represents the average RPKM for the six individuals of each treatment (C = control; HS = heat stress; HSS = heat stress + starvation; S = starvation). Rows (genes) and columns (treatments) are clustered hierarchically based on their expression values. Color scale reflects row scaling that was performed in the R package *pheatmap* to aid in visualization.

## Description

As climate change progresses, most marine species will experience multiple stressors simultaneously (Chevin, 2013; Etterson & Shaw, 2001) . It is imperative to better understand the molecular pathways responding to these multiple environmental stressors if we want to accurately predict whether species will be able to survive changing conditions. Importantly, marine invertebrates have varied responses to concurrent stressors. For example, exposure to some stressors simultaneously results in trade-offs that reduce tolerance (Kelly et al., 2016) . In corals, exposure to heat stress on its own results in downregulation of calcification genes (Barshis et al., 2013; Kenkel et al., 2013) ; however, exposure to low pH, which is occurring along with rising temperatures in many oceans, causes the opposite response: an *upregulation* of these genes (Moya et al., 2012) . In other situations, tolerance to one stressor can help prime the organism to tolerate an additional stressor, as has been observed in warm-acclimated *Echinogammarus marinus* amphipods that also show higher performance in hypoxia conditions (Collins et al., 2021a). A similar cross-tolerance effect has also been observed in *Tigriopus californicus* copepods that have higher thermal tolerance after being exposed to elevated salinity (Denny & Dowd, 2022) . Lastly, other combinations of stressors can lead to unique responses not observed with either singular stressor. In sea urchin larvae exposed to ocean acidification and heat stress concurrently, metabolic genes were downregulated, even though these same genes did not respond to either stressor on its own (Padilla-Gamiño et al., 2013). In each of these examples, knowledge of single stressor gene expression responses does not allow us to accurately predict the response to multiple concurrent stressors. Thus, only multi-stressor studies can provide an accurate assessment of transcriptome-wide gene expression responses to the suite of stressors marine invertebrates experience in the field.

Two stressors that are occurring simultaneously for many marine invertebrates are high temperatures and low food conditions. For example, off the California coast of western North America, the economically important red abalone *Haliotis rufescens* is experiencing not only high temperatures due to an increased frequency of warm water El Niño events, but also starvation conditions. The warm waters associated with El Niño events carry less nutrients, and thus bull kelp *Nereocystis leutkeana* , the primary food source of *H. rufescens,* has been reduced by up to 93% in some areas (Rogers-Bennett & Catton, 2019). Notably, climate change is expected to double the frequency of these El Niño events (Cai et al., 2014) ; thus, understanding how *H. rufescens* responds to these simultaneous stressors is essential if the $44 million per year recreational fishing industry is to be preserved (Reid et al., 2016) .

In this experiment, we used RNA-sequencing to assess the gene expression response to combined thermal and starvation stress in the red abalone *Haliotis rufescens.* Juvenile *H. rufescens* were exposed to four separate treatments for 14 days: 1) control conditions, 2) heat stress conditions, 3) starvation conditions, and 4) heat stress + starvation conditions. RNA from six individuals per treatment group (24 total samples) was extracted using a standard Tri-Reagent protocol and sequenced using Illumina PE150 technology. Cleaned reads were mapped to the *H. rufescens* genome (Masonbrink et al., 2019) to obtain gene annotation information and gene expression counts for each contig in the transcriptome. We used Principal Component Analysis (PCA) and the R package DESeq2 to assess global transcriptomic responses to each treatment and to identify significantly differentially expressed genes between treatment groups, respectively. Lastly, the R package topGO was used to perform Gene Ontology (GO) enrichment analyses on each identified group of differentially expressed genes.

Changes in gene expression were observed in all three stress treatment groups, as visible in the PCA in Figure 1A . Specifically, Principal Component 2 (PC2) separates the control group from all other treatment samples. Regarding differential expression, 341 genes responded to all three experimental conditions ( Figure 1B ; Extended Data File 1). The top biological processes significantly enriched in this shared response gene group are nucleosome assembly (Fisher’s test p = 0.00011), protein-DNA complex assembly (p = 0.00011), nucleosome organization (p = 0.00011), protein-DNA complex organization (p = 0.00015), and chromatin organization (p = 0.00015) and remodeling (p = 0.00015; Extended Data File 2).

The amount of overlap in gene expression response, i.e., genes that responded to more than one treatment, varied. Twenty-one total genes responded to both heat stress and starvation single stressor conditions ( Figure 1B ). Nine of these genes were upregulated in both treatments, including one cytochrome P450 that plays a role in oxidative metabolism (Rewitz et al. 2006) . Six of these genes were downregulated in both treatments. Downregulated genes include two membrane-related genes: ionotropic glutamate receptor and Vang-like protein, a planar cell polarity protein (Extended Data File 1). 808 genes responded to both heat stress and heat stress + starvation conditions; 415 genes were upregulated, and 393 were downregulated. Upregulated genes are significantly enriched for two GO terms related to transcription (including 3 leucine-zipper domain and 3 AP-1 transcription factor genes) and two GO terms related to protein folding chaperones (including 2 heat shock protein [hsp] 70s and 2 HSP90s; Figure 1C ). Downregulated genes are significantly enriched for RNA binding (p = 4.20E-06; Extended Data File 2) and include 11 RNA recognition motif domain genes (Extended Data File 1). 873 genes responded to starvation and heat stress + starvation conditions. 368 genes were upregulated and were not enriched for any GO terms; however, this group did include 19 genes involved in the ubiquitin/proteosome pathway, such as 7 WD40 repeats and 4 F-box domains ( Figure 1D ). 505 downregulated genes were significantly enriched for the biological process of cell adhesion; these genes include 6 ependymins, a glycoprotein that has also been observed in marine sponges (Kawasaki et al., 2023) , sea urchins, and sea cucumbers (Suárez-Castillo et al., 2004), but whose physiological function remains largely unknown.

Despite some genes responding to multiple treatments, the PCA clearly separates the three stress treatment groups into distinct non-overlapping clusters ( Figure 1A ), indicating that *H. rufescens* has a unique transcriptomic response to each environmental stressor in multivariate space. Regarding genes differentially expressed only in response to a single treatment, heat stress had the most muted and least unique response of the three treatments: 337 genes (22.4% of the total number of genes that changed expression in response to this stressor) responded only to heat stress ( Figure 1B ; Extended Data File 1). 145 upregulated genes were not significantly enriched for any GO terms, but did contain 7 ankyrin repeat genes involved in protein binding. 192 downregulated genes were significantly enriched for sequence-specific DNA binding (p = 0.0048), including 2 forkhead domain and 1 Ets domain genes. 849 genes (40.7% of the total number of differentially expressed genes under starvation) responded only to starvation conditions: 323 of these genes were upregulated and 526 were downregulated. The most enriched GO term in the upregulated genes was ribonucleoprotein biogenesis (p = 0.0048), although only two genes in this category were annotated (Extended Data File 1). For the downregulated genes, the most significantly enriched molecular function was unfolded protein binding (p = 2.20E-06); genes representing this GO term included 2 HSP90s, 2 chaperonins, and 1 calreticulin/calnexin gene ( Figure 1C ). Two additional related GO terms of protein folding chaperone (p = 0.00321) and ATP-dependent protein folding chaperone (p = 0.00321), represented by two HSP70s, were also enriched in the downregulated genes. The most significantly enriched biological process was transmembrane transport (p = 6.40E-05): 6 sodium/solute symporters and 6 major facilitator genes that function in nutrient uptake (Reizer et al., 1994; Marger & Saier, 1993) were downregulated ( Figure 1E ). Lastly, the multi-stressor heat stress + starvation treatment induced the largest, and most unique, gene expression response compared to either stressor alone. 1,966 genes (49.3% of the total number of genes that responded to this treatment) changed expression *only* under heat stress + starvation conditions. Unlike with starvation and heat stress single stressors, in this multi-stressor treatment a higher number of genes was upregulated (1023) compared to downregulated (943). Upregulated genes are most enriched for the biological process of translation (p < 1e-30), peptide biosynthetic process (p < 1e-30), and amide biosynthetic process (p < 1e-30; Extended Data File 2). Regarding translation, 20 large ribosomal subunit proteins and 19 small ribosomal submit proteins were all upregulated in the heat stress + starvation condition compared to controls ( Figure 1F ). Downregulated heat stress + starvation genes are most enriched for the molecular function nucleotide binding (p = 0.00025), including 2 aminoacyl-tRNA synthetases and 2 Gfo/Idh/MocA-like oxidoreductases (Extended Data File 1).

This study found that the transcriptome-wide response to the combined stressors of heat stress + starvation is largely characterized by an upregulation of ribosomal subunit proteins involved in translation. These findings are consistent with previous studies that suggest translation is upregulated under stressful conditions in some marine invertebrates. In the coral *Acropora tenuis* translation and ribosome biogenesis genes are expressed more highly in individuals from poor water quality (i.e., more stressful) environments (Rocker et al., 2019) . In *Crassostrea gigas* Pacific oyster larvae exposed to concurrent low pH, high temperature, and low salinity, an upregulation of proteins involved in ribosome structure was observed (Dineshram et al., 2016) . Our results also support previous multi-stressor research in other marine mollusks such as mussels. For example, *Mytilus galloprovincialis* mussels exposed to both heat stress and nickel simultaneously (Mohamed et al., 2014) or heat stress and copper simultaneously (Negri et al., 2013) upregulated ribosome biogenesis genes involved in translation. These authors hypothesized that such upregulation was a compensatory strategy to accommodate the increased protein synthesis needed to tolerate multiple stressors. Red abalone in this study might be using a similar strategy to cope with sublethal heat stress + starvation conditions.

Heat shock proteins (HSPs) involved in unfolded protein binding were significantly upregulated in heat stress and heat stress + starvation conditions, but were significantly downregulated in starvation conditions. Similar to our results, several HSPs were also upregulated in the coral *Oculina arbuscula* under both fed, heated and unfed, heated conditions (Rivera et al., 2023) . Overall, HSP90s that bind to denatured proteins and refold them to their proper conformation are also upregulated under heat stress conditions in a wide variety of other marine invertebrates, including the Pacific abalone *Haliotis discus hannai* (e.g., Chen et al., 2019; Kyeong et al., 2019; Wu et al., 2023). HSPs are also known to play an important role in multi-stress resistance in plants (Jacob et al., 2017) . The downregulation of HSPs we observed in starvation individuals contrasts previous work in the soft coral *Paramuricea clavata* that found an increase of HSP70 and HSP90 protein expression when food availability is low (Rossi et al., 2006) . However, other invertebrate studies in starved *Musca domestica* housefly larvae and *Daphnia pulex* microcrustaceans reported reduced HSP expression (Tian et al., 2018) and calreticulin protein expression (Becker et al., 2018) , respectively, which matches our results. Importantly, the fact that unfolded protein binding genes show opposite patterns of expression in heat stress and starvation conditions highlight the existence of energetic tradeoffs during stress and could reflect low energy availability in the starved individuals (Sokolova, 2013) . HSPs use ATP to refold damaged proteins and are thus energetically costly; for example, the marine mollusk *Crassostrea virginica* exposed to heavy metals spends ~40% of its total ATP supply on the production and functioning of stress proteins such as HSPs (Cherkasov et al., 2006; Ivanina et al., 2008) . Similarly, the downregulation of unfolded protein binding genes we observed in the starvation treatment could indicate a larger need to downregulate ATP-consuming pathways to facilitate survival under low nutrient availability (Sokolova et al., 2012) . The fact that the GO terms ATP metabolic process (p = 0.00034), ATP biosynthetic process (p = 0.00594), and proton-motive force driven ATP synthesis (p=0.00594) are also enriched in the genes downregulated only in the starvation treatment further support this hypothesis (Extended Data File 2).

In the ubiquitin stress response pathway, proteins irreversibly damaged are degraded (Parag et al., 1987) . In this study genes involved in the ubiquitin/proteosome pathway, including 7 WD40 repeats and 4 F-box domains that function as substrate receptor subunits of the SCF ubiquitin-ligase complex (Xu & Min, 2011; Kipreos & Pagano, 2000) , were significantly upregulated under starvation conditions. Previous data on the ubiquitin response to nutrient limitation is mixed: starvation downregulates the ubiquitin proteosome pathway in *Oncorhynchus mykiss* rainbow trout (Martin et al., 2022) , but polyubiquitin transcript levels increased in food-deprived *Sepia officinalis* cuttlefish (Lamarre et al., 2016) . Our results also indicate increased expression of WD40 and F-box domain ubiquitin-associated genes under heat stress + starvation conditions, which matches previous multi-stressor work in marine mussels: *Mytilus galloprovincialis* exposed to concurrent nickel and temperature stress also upregulated proteolysis-related genes (Mohamed et al., 2014) . As noted above, molecular chaperones such as heat shock proteins refold denatured proteins; however, if the stress-induced damage to the protein is irreversible, the protein can’t be repaired and instead must be degraded through the ubiquitin/proteosome pathway to maintain protein homeostasis (Kandel et al., 2015) . Thus, an upregulation of ubiquitin stress response genes in the starvation and heat stress + starvation treatments, but not the heat stress treatment, could indicate a higher degree of stress, and resulting physiological damage, in the starvation and heat stress + starvation treatments. For example, in the marine snail *Tegula funebralis,* northern California individuals that are less thermally tolerant show a higher upregulation of ubiquitin-associated genes than more thermally tolerant southern California individuals after heat stress (Gleason & Burton, 2015) .

Transmembrane transporters involved in nutrient transport, including 6 sodium/solute symporters and 6 major facilitator genes, were significantly downregulated only in the starvation treatment. (Although there is evidence of lowered expression of these transporters in the heat stress + starvation treatment, there is variation among individuals and overall this expression change is not significant.) Downregulation of membrane transport proteins has also been observed in aestivating, non-feeding *Apostichopus japonicus* sea cucumbers (Chen et al., 2016) . In contrast, previous work in nitrogen-starved *Tetraselmis suecica* green algae (Lauritano et al., 2019) and starved *Drosophila* fruit flies (Park et al., 2016) found an increase in solute transport transcripts. Overall, the downregulation of transmembrane transporters we observed in red abalone under starvation conditions, similar to the downregulation of HSPs discussed above, could reflect an energy-saving strategy. Sodium/solute symporters and members of the major facilitator superfamily are secondary active transporters (Drew et al., 2021); thus, a downregulation of these genes under low food conditions when there are no nutrients to transport could conserve energy that can instead be used for other processes essential for survival.

In this paper we conclude that the genes responding to heat stress, starvation, and heat stress + starvation in red abalone are largely unique. However, we note that this study assessed differential expression of transcripts, but did not examine protein expression levels. Thus, it is unknown if the changes we observed in transcription across treatments also resulted in changes in translation. Notably, in other marine mollusks such as *Mytilus californianus,* there is evidence that transcription and translation are often uncoupled (Gleason et al., 2023) . It is also worth emphasizing that individuals exposed to heat stress conditions in this study experienced a slow ramping rate of +2 °C per 5 days. This ramping rate could have been slow enough to allow for some acclimatization of individuals, and thus could indicate that the gene expression changes we observed in response to heat stress are muted compared to what would be observed with faster thermal ramping rates (e.g., Collins et al., 2021b). Lastly, another important caveat to keep in mind is that these experiments were performed on small juvenile abalone, which are known to be more sensitive to stressors such as heat (Steinarsson & Imsland 2003; Rogers-Bennett et al., 2016) . Thus, caution should be used when applying these results to red abalone at different developmental stages.

These results highlight the importance of multi-stressor studies that more accurately represent conditions in the field, where individuals are exposed to multiple changing environmental conditions simultaneously. Our results indicate that ~2000 genes are differentially expressed only under combined heat stress + starvation conditions and are characterized by a significant upregulation in translation genes. This suggests that exposure to both stressors at the same time produces a compounded effect that is not simply the sum of the responses to each individual stressor. Moreover, because the gene expression responses to heat stress and starvation are largely unique, and in some cases induce opposing gene expression responses, it is unlikely that response to one stressor will provide tolerance to the other. Overall, this study emphasizes that accurate predictions of transcriptomic responses, and ultimately climate change impacts, will require multi-stressor studies.

## Methods

Juvenile *Haliotis rufescens* 10-15mm in shell length were obtained from two different aquaculture farms: Monterey Abalone Company (MAC) in Monterey, California and the Cultured Abalone Farm (CAF) in Goleta, California. Monterey Abalone Company originally obtained wild broodstock from Timber Cove, California in 2012, and the Cultured Abalone Farm obtained broodstock from the Santa Barbara Channel between 1995 and 2010. Juvenile *H. rufescens* were acclimated to common garden conditions (~15 °C, *ad libitum* dried algae food) for three weeks. Using established fecal collection protocols from the California Department of Fish and Wildlife Shellfish Health Laboratory in Bodega Bay, California and PCR primers targeting the 16SrRNA gene of the bacteria *Candidatus Xenohaliotis californiensis* (Andree et al., 2000) , we determined that no juveniles had withering syndrome, a common wasting disease in abalone that could confound later results.

Following common garden acclimation, juveniles were split into four separate groups: 1) starvation (S); 2) heat stress (HS); 3) heat stress + starvation (HSS); and 4) control (C) conditions. Starvation individuals were fed 10% of normal *ad libitum* food intake. Heat stress individuals were exposed to warm water El Niño-like temperatures (16°C on Day 1, 18°C on Day 5, 20°C on Day 10, and 21°C on Day 14). Heat stress + starvation individuals were given 10% of normal food intake and subjected to El Niño-like temperatures. Control individuals were kept at normal conditions (15 °C and *ad libitum* food). All juveniles were exposed to experimental conditions for 14 days, which is a time period sufficient to cause stress, but not to cause mortality.

At the end of the experimental period, 12 abalone from each treatment group were flash frozen in liquid nitrogen. RNA was extracted from whole body tissue using a standard Tri-Reagent protocol. The six individuals (3 from each farm) from each of the four treatment groups (24 total individuals) with the highest RNA concentration and purity ratios were sent to the UC Davis Genome Core for library preparation and Illumina PE150 sequencing.

CLC Genomics Workbench (version 21.0.4) was used for quality control and read mapping of sequences. First, sequences were trimmed for ambiguity (a maximum of two ambiguous nucleotides were allowed) and quality (low quality reads below a 0.025 limit were removed). Reads shorter than 60 bp were discarded. Reads were then mapped to the 57,784 contigs of the *Haliotis rufescens* genome (Masonbrink et al., 2019) using a minimum fraction length of read overlap = 0.8 and a minimum sequence similarity = 0.95. Uniquely mapped reads, along with their associated gene annotations from the published *H. rufescens* genome, resulted in 45,965 contigs for use in subsequent analysis. In all subsequent analyses samples from the two different abalone farms were grouped together; thus, the results provide an assessment of a more general *H. rufescens* response to high temperatures, low food conditions, and high temperature plus low food conditions, respectively.

The reads per kilo base of exon model per million mapped reads (RPKM) values for each gene, as calculated by CLC Genomics Workbench during mapping, were used to assess overall differences in the transcriptomic response to the various environmental conditions in multivariate space. A Principal Component Analysis (PCA) was performed on these normalized RPKM expression values of all samples using the R package FactoMine (Lê et al., 2008) and visualized with the R package factoextra (Kassambara & Mundt, 2020) .

Uniquely mapped read counts were further analyzed using the R package DESeq2 (Anders & Huber, 2010) to identify differentially expressed genes among treatment groups. Reads were normalized for library size, and contigs with average normalized expression <5 were excluded from analyses. For differential expression analyses a false discovery rate of 5% was used (Benjamini & Hochberg, 1995) . Three pairwise comparisons were performed to examine gene expression differences in response to the experimental conditions: 1) control vs. heat stress samples; 2) control vs. starvation samples; and 3) control vs. heat stress + starvation samples.

Identified groups of differentially expressed genes were tested for functional enrichment of Gene Ontology (GO) categories using the R package topGO (Alexa & Rahnenfuhrer, 2024) . All 45,965 genes expressed in at least one treatment in this study were used as the background set of genes for these comparisons. Seven different gene sets of interest were tested for functional enrichment: 1) genes differentially expressed (DE) in heat stress (HS), starvation (S), and heat stress + starvation (HSS) conditions; 2) genes DE in HS and S conditions; 3) genes DE in HS and HSS conditions; 4) genes DE in S and HSS conditions; 5) genes DE only in HS conditions; 6) genes DE only in S conditions; and 7) genes DE only in HSS conditions. For each set of genes, the three GO attributes of molecular function, biological process, and cellular component were analyzed. Functional enrichment tests for each GO attribute of each gene set of interest were performed using the ‘classic’ algorithm and the Fisher’s test statistic, using a conservative significance value of 0.01. RPKM expression values for genes of interest in select functionally enriched GO categories were visualized with the R package pheatmap (Kolde, 2018) using row scaling.

## Data Availability

Description: Genes identified to be significantly differentially expressed for each component of the Venn diagram in Figure 1B. The annotation information, RPKM expression value for each individual in each of the four treatments, and the average RPKM expression value per treatment are provided for each gene. . Resource Type: Dataset. DOI: https://doi.org/10.22002/n0y4x-xx706 Description: Significantly enriched Gene Ontology (GO) terms identified by topGO for each component of the Venn diagram in Figure 1B.. Resource Type: Dataset. DOI: https://doi.org/10.22002/wqgkp-tnd16
